# Gastrointestinal parasites in captive olive baboons in a UK safari park

**DOI:** 10.1017/S0031182023000823

**Published:** 2023-10

**Authors:** Alexandra Juhasz, Elly Spiers, Ellie Tinsley, Emma Chapman, William Shaw, Marion Head, Lucas J. Cunningham, John Archer, Sam Jones, Lee R. Haines, Naomi Davies Walsh, Bridget Johnson, Jen Quayle, Jayne Jones, Elwyn James LaCourse, Jonathan Cracknell, John Russell Stothard

**Affiliations:** 1 Tropical Disease Biology, Liverpool School of Tropical Medicine, Liverpool L3 5QA, UK; 2 Institute of Medical Microbiology, Semmelweis University, H-1089 Budapest, Hungary; 3 Research and Conservation, Knowsley Safari, Prescot, Merseyside L34 4AN, UK; 4 Clinical Sciences, Liverpool School of Tropical Medicine, Liverpool L3 5QA, UK

**Keywords:** giardiasis, *Papio anubis*, strongyloidiasis, *Strongyloides fuelleborni*, trichuriasis, *Trichuris trichiura*

## Abstract

From the safety inside vehicles, Knowsley Safari offers visitors a close-up encounter with captive olive baboons. As exiting vehicles may be contaminated with baboon stool, a comprehensive coprological inspection was conducted to address public health concerns. Baboon stools were obtained from vehicles, and sleeping areas, inclusive of video analysis of baboon–vehicle interactions. A purposely selected 4-day sampling period enabled comparative inspections of 2662 vehicles, with a total of 669 baboon stools examined (371 from vehicles and 298 from sleeping areas). As informed by our pilot study, front-line diagnostic methods were: QUIK-CHEK rapid diagnostic test (RDT) (*Giardia* and *Cryptosporidium*), Kato–Katz coproscopy (*Trichuris*) and charcoal culture (*Strongyloides*). Some 13.9% of vehicles were contaminated with baboon stool. Prevalence of giardiasis was 37.4% while cryptosporidiosis was <0.01%, however, an absence of faecal cysts by quality control coproscopy, alongside lower than the expected levels of *Giardia*-specific DNA, judged RDT results as misleading, grossly overestimating prevalence. Prevalence of trichuriasis was 48.0% and strongyloidiasis was 13.7%, a first report of *Strongyloides fuelleborni* in UK. We advise regular blanket administration(s) of anthelminthics to the colony, exploring pour-on formulations, thereafter, smaller-scale indicator surveys would be adequate.

## Graphical abstract

**Figure:**
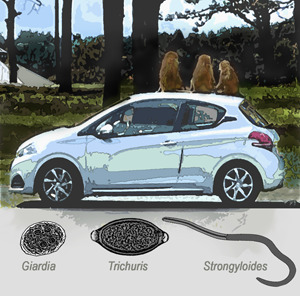

## Introduction

**Figure 1.**
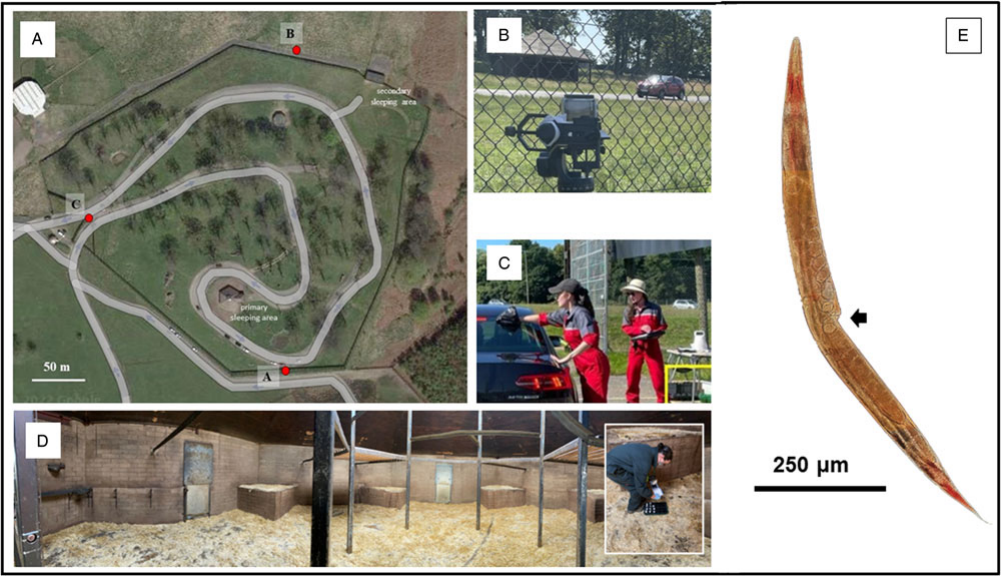
KS baboon enclosure with selected activities illustrated as undertaken during the 4-day sampling period. (A) An aerial image of the enclosure with tarmac road highlighted, the positions of the 3 remote video cameras around the baboon enclosure (red dots) are shown at position A (in view of primary sleeping area), at position B (in view of secondary sleeping area) and at position C (in view of vehicle entrance and exit). (B) A view of CamB, typical of the other cameras, which was used to capture baboon–vehicle interactions, as played back in slow motion, during the video analyses. (C) Stool samples collected from the roof of a car using a gloved-hand and a plastic bag. The vehicle licence plate, the position of the stool and the time of entrance/exit was each recorded. (D) A panoramic image of inside the primary sleeping area. Stool samples were collected from the cement-lined floor (inset) using a gloved-hand and a plastic bag. (E) A light micrograph of an adult female worm of *Strongyloides fuelleborni*, stained in Lugol's iodine; note the characteristic vulvar region (black arrow) that permits morphological differentiation from *Strongyloides stercoralis*. This worm was retrieved upon an additional charcoal culture exercise as undertaken in October 2022.

Knowsley Safari (KS) is situated in the northwest of England, Merseyside (53.4339° N, 2.8126° W) on the Earl of Derby's estate. It first opened to the public in 1971. Today, KS is both a member of the British and Irish Association of Zoos and Aquariums and the European Association of Zoos and Aquaria. It is home to over 750 exotic animals and extends across 220 hectares of land. Each year, KS attracts around 600 000 paying visitors and runs extensive onsite public education campaigns about wildlife conservation and management. Its most popular enclosure on the safari drive is the baboon enclosure (Lloyd *et al*., 2021). Here, around 240 captive baboons reside within a ring-fenced 6.4-hectare compound, with 2 roofed sleeping areas, and 845 m of circular tarmac road (Fig. 1A).

The KS colony is presumed mostly of olive baboon (*Papio anubis*) stock. Its germinal origins, however, included hamadryas (*Papio hamadryas*), chacma (*Papio ursinus*) and yellow baboon (*Papio cynocephalus*) as well as drills (*Mandrillus leuophaeus*). This animal mixture was initially provided by Jimmy Chipperfield, former co-director of Chipperfield's Circus, for the opening of the safari. Presently, the baboons have become a flagship attraction for KS as, from the safety of their own vehicles or a KS public bus, visitors can get up close to these animals. This permits a unique baboon viewing experience within their semi-natural habitat. Of note, as baboons regularly mount and explore vehicles, they may also defecate onto metal and glass surfaces. Over and above standard guidance in baboon stool disposal, the unknown status of zoonotic parasites within the colony itself elicits a public health concern.

Gastrointestinal parasites of various baboon species living in wild, or in conservation settings, are well documented over decades of study using necropsy and coprological surveys (Myers and Kuntz, 1965; Murray *et al*., 2000; Hahn *et al*., 2003; Hope *et al.*, 2004; Fagiolini *et al*., 2010; Mafuyai *et al*., 2013; Ebbert *et al*., 2015; Reichard *et al*., 2017; Akinyi *et al*., 2019; Eo *et al*., 2019; Mbuthia *et al*., 2021). The gastrointestinal parasites of olive baboons have been frequently studied in different countries, sometimes with DNA characterization of encountered parasites (Ko *et al*., 2023), to assess their zoonotic potentials (Müller-Graf *et al*., 1996; Munene *et al*., 1998; Bezjian *et al*., 2008; Ryan *et al*., 2012; Larbi *et al*., 2020; Tabasshum *et al*., 2022) that might have bearing for this UK safari park setting.

While the status of gastrointestinal parasites of public health importance within the KS baboon colony is unknown, in 2019 an adult female baboon underwent a post-mortem investigation where numerous whipworms were found within its caecum and large bowel. Upon the request of Dr Jonathan Cracknell, KS, Head of Living Collections, these whipworms were later identified and genotyped at the Liverpool School of Tropical Medicine, confirming the presence of the human whipworm *Trichuris trichiura*. This observation sparked the later request to conduct a more comprehensive parasitological assessment of the baboon colony, particularly as a pilot DNA screen of several baboon stools with real-time polymerase chain reaction (PCR) diagnostics revealed the presence of *Strongyloides*.

The report presented here forms the main part of an initial public health and animal welfare investigation. Our primary objective was to qualify and quantify key gastrointestinal parasites of public health concern within baboon stool found on contaminated vehicles, taking advantage of ‘clean catch’ from glass and metal surfaces. Our secondary objective was to inspect the baboon colony itself, using stools more readily collectable from its 2 cement-lined sleeping areas, which were regularly cleaned, as a more general and comparative parasitological screen.

## Materials and methods

### Study sampling design and stool specimen collection

After consultations with KS management and administration staff, in July of 2021, four 1 day surveys, designated survey *S**a*** (on 14.07.2021), *S**b*** (on 17.07.2021), *S**c*** (on 28.07.2021) and *S**d*** (on 31.07.2021)] were selected for the baboon stool collection during KS opening times. This permitted comparisons between low-season (Sa and Sb) and high-season (Sc and Sd) KS visitor admissions. Survey Sa was conducted during mid-week/low-season, survey Sb was weekend/low-season, survey Sc was mid-week/high-season and survey Sd was weekend/high-season. Low- and high-seasons corresponded to school attendance or school holiday times during the summer period which each creates clear differences in KS visitor attendance numbers.

Baboon stools were collected from 2 major sampling zones: first, from the exterior of visitor's vehicles (Fig. 1C) and second, from each primary and secondary sleeping areas within the baboon compound (Fig. 1A and D). Since directly observed stool collection from individual baboons was not feasible, all samples were taken from anonymous deposition. Given the total number of baboons was 231 individuals at the time of last census in the preceding year, our stool sampling target was to have parable numbers of stool collected as the number of baboons, each replicated for the 2 sampling strategies, i.e. from vehicles and from sleeping areas. This split design approach enabled a formative comparison between public health risk ‘from the colony’ and parasite endemicity ‘within the colony’. Our ‘clean-catch’ strategy from vehicles reduces contamination from ground-dwelling nematodes and, while having a direct daily comparison of collected stools from vehicles vs sleeping areas, aimed to qualify and quantify public health risk.

### Stool specimen collection from vehicles

For each day of stool collection, approximately 7 h of sampling was set aside for obtaining stool from vehicles that had driven through the baboon enclosure. Here, the registration number, entrance and exit times of each vehicle were noted, alongside vehicle type (car, van or minibus) as well as current weather conditions (sunny, cloudy or rainy). At the enclosure exit, each vehicle was briefly visually inspected for baboon stool by 3 observers; if present, stool was hand-picked by gloved-hand, then wrapped inside a plastic bag and labelled with a unique ID code. This code was relatable back to the number of stools on the vehicle and their deposition locations (bonnet, windshield, roof). At the completion of daily sampling, stools were taken to the field laboratory for onsite coprological examinations the following day (see below).

### Stool specimen collection from sleeping areas

On each survey day, an hour in the morning, before the baboon enclosure opened, was set aside for inspection of the 2 sleeping areas for fresh stool. The previous day, KS keepers had cleaned each area and laid down fresh bedding straw. For the purpose of the survey, the sleeping areas were split into 3 sections of roughly equal size, where 25–30 stools were hand-picked by gloved-hand from the cement-lined floors in each area, then wrapped inside a plastic bag (Fig. 1D). Each stool sample was labelled with a unique ID code relating to its location and date, and then taken to the field laboratory for onsite coprological examinations the following day (see below).

### Video capture of vehicles and baboons

To gain an insight into the baboon–vehicle behaviours, 3 fixed-point remote video recordings of the enclosure were captured during each survey with digital video cameras (AKASO EK7000 Pro 4K Action Camera). The 3 cameras were designated CamA, CamB and CamC, respectively, and placed on tripods outside of the baboon compound: *location A* – in view of the primary sleeping area, *location B* – opposite to the secondary sleeping area and *location C* – between the vehicle entrance and exit (Fig. 1A). A typical field of view from a camera is shown (Fig. 1B).

Each camera was placed on continuous wide-angle, high-precision video record mode; however, its 1050 mAh rechargeable battery needed replacement every 40–45 min, from a pool of pre-charged battery stocks. The time-stamped video footage of camera CamC at location C also enabled verification of vehicle entrance and exit times, corroborating the duration that each vehicle spent within the enclosure as calculated upon direct observation and paper record reporting.

Video footage from the 3 cameras was further reviewed, by slow motion play back, to gather estimate information on the number of adult males, adult females and juvenile baboons mounting vehicles throughout the day. Upon assessment, video footage from survey Sd alone was used to assess for any temporal associations between the number of stools and contaminated vehicles. Surveillance video from survey Sd was judged most successful, as it captured the longest continuous duration of video footage (~7 h), as battery replacements were best achieved, and inclement weather was minimal. An 8-min video recording from inside a vehicle driving slowly through the baboon enclosure is provided (see online materials) with YouTube upload for viewing (https://www.youtube.com/watch?v=jxqg0wsSITA).

### Coprological analysis methods

As guided from our pilot inspection, 3 ‘front-line’ diagnostic methods were selected to ascertain the prevalence of key gastrointestinal parasites: QUIK-CHEK RDT (TechLab Inc., Blacksburg, Virginia, USA) to detect *Giardia* and *Cryptosporidium*, coproscopy by Kato–Katz to detect *Trichuris* (Tarafder *et al*., 2010; Rivero *et al*., 2020) and charcoal culture incubation to detect *Strongyloides* (Dancescu, 1968). The QUIK-CHEK assay has been shown to have diagnostic sensitivity and specificity for *Giardia* and *Cryptosporidium* of 93.3 and 99.4%, and 87.6 and 98.9%, respectively for human infections (Van den Bossche *et al*., 2015). Two ‘second-line’ coprological methods of sedimentation and floatation of stool were used in corroboration of front-line methods as quality control, permitting detection of other gastrointestinal parasites.

#### Front-line method: coprology by RDT

The *Giardia*/*Cryptosporidium* QUIK-CHEK RDT was carried out according to the manufacturer's protocol with all stool samples. To gain estimate of the likely number of *Giardia* cysts present, a serial dilution series of 2 very strongly positive samples was conducted.

#### Front-line method: coproscopy by Kato–Katz

Two Kato–Katz thick smears (2 × 41.7 mg) were prepared per sample on the same microscope slide and were each examined using a compound light microscope (Katz *et al*., 1972). Egg counts were tallied for *Trichuris* while any other parasitic ova, larvae or cysts identified were noted. The eggs per gram (EPG) of faeces were calculated to categorize the intensity of *Trichuris* infection with thresholds of: *light* (1 to ⩽999), *moderate* (1000 to ⩽9999) and *heavy* (⩾10 000) (see WHO, 1991).

#### Front-line method: coproscopy by charcoal culture

From our pilot observation of first indication for *Strongyloides* spp., baboon stool was subjected to charcoal culture (Dancescu, 1968; Yelifari *et al*., 2005). About 3 g of fresh stool was mixed with an equivalent of powdered charcoal in a Petri dish, and then incubated for 5 days at ~20°C within a heated polystyrene box. After incubation, if larvae or adult worms were viewed under the stereomicroscope, a selection was then removed by pipette, stained with Lugol solution on a glass slide and viewed under a compound microscope at ×400 magnification. A selection of isolated larvae was placed in 95% ethanol for species identification using PCR (see below).

#### Second-line method: coproscopy by sedimentation and flotation

A 10% formalin-fixed stool specimen archive, taken randomly from those samples deemed positive for giardiasis by QUIK-CHEK RDT, was examined by sedimentation upon centrifugation with the formalin–ether concentration method following WHO (1991). The samples were also inspected by floatation with centrifugation using a zinc sulphate solution at a specific gravity of 1.2 (Zajac *et al*., 2002). Each method was performed at the Liverpool School of Tropical Medicine with slide preparations viewed under ×400 light microscopy.

### Statistical analyses

Observed data pertaining to vehicles entry and exit times, and contamination with stool, were recorded in field notes, then transcribed, upon double entry, using Excel ver. 2211 Microsoft 365 Apps for enterprise. Tallies of helminth ova were first recorded in field notes, then transcribed, upon double entry, using Excel. Data trends and geometric EPGs were first inspected by cross-tabulation, then upon visual inspection of graphical analyses, with 95% confidence intervals (CIs) assigned. Any differences in prevalence between ‘clean-catch’ vs ‘sleeping area’ were compared using *Z*-scores of observed proportions.

### Molecular DNA characterization of *Giardia*

To corroborate RDT findings, total DNA was isolated from each RDT positive stool sample using the QIAamp DNA mini kit (Qiagen, Hilden, Germany) according to the manufacturer's instructions, with minor revisions including a bead-beating step as detailed previously (Minetti *et al*., 2015). A nested PCR was carried out to detect and amplify a 511-bp region of the *Giardia duodenalis β*-giardin (*bg*) gene for Sanger sequencing and genotyping according to Al-Shehri *et al*. (2018). Nested-PCR products were purified using the QIAquick PCR purification kit (Qiagen) according to the manufacturer's instructions and then were sequenced in both directions with PCR primers using Sanger sequencing (Al-Shehri *et al*., 2018). The obtained chromatograms were visualized and nucleotide sequences were trimmed/masked and edited using MEGA X version 10.2.6 (Kumar *et al*., 2018).

Assemblages were identified by nucleotide basic local alignment search tool (BLAST) searches within the NCBI database using each sequence in turn against the *G. duodenalis* species-specific (non-assemblage-specific) database. Nucleotide BLAST hits with the highest match and query cover scores as well as lowest *E*-values were considered the most closely related assemblage and the sequence of each most closely related BLAST hit was downloaded from GenBank in the FASTA format to serve as an assemblage-specific reference sequence. To identify any samples potentially co-infected with multiple *G. duodenalis* assemblages, a reference sequence of each assemblage A–H was also downloaded from GenBank in the FASTA format.

### Molecular DNA characterization of *Trichuris*

During autopsy of a baboon adult during these surveys, adult *Trichuris* worms were isolated from the caecum and preserved in absolute ethanol. Three individual worms were then selected and underwent genomic DNA extraction using the QIAamp DNA mini kit (Qiagen) according to the manufacturer's instructions, but inclusive of a pre-treatment bead-beating step using ~0.9 g of 1.4 mm ceramic beads at 3000 rpm for 30 s within a MagNa Lyser (Roche Diagnostics, Rotkreuz, Switzerland).

Purified genomic DNA then underwent PCR amplification using the forward 18S965F (5′GGCGATCAGATACCGCCCTAGTT3′) and reverse 18S1573R (5′TACAAAGGGCAGGGACGTAGT3′) PCR primers (Guardone *et al*., 2013). Reactions consisted of 5 *μ*L of template DNA (20 ng *μ*L^−1^) in 25 *μ*L containing 12.5 *μ*L MyTaq™ Red Mix (Bioline, London, UK), 400 nm of each primer adjusted with nuclease-free water. The thermal PCR amplification consisted of an initial denaturation step at 95°C for 1 min, followed by 40 cycles of denaturation at 95°C for 15 s, annealing at 59°C for 15 s and extension at 72°C for 30 s. After the 40th cycle, there was an additional extension step of 72°C of 2 min. Amplicons were separated in a 1.2% agarose gel for 1 h at 100 V and then stained with Invitrogen™ SYBR™ Safe DNA Gel Stain (Fisher Scientific, Loughborough, UK).

Amplicons were sent for Sanger sequencing at Source Bioscience, UK using both forward and reverse primers, generating a 689 bp internal sequence, identical across all 3 worms (accession number: OR395368). This sequence was then aligned against reference sequences from the *Trichuris* using the basic local alignment search tool (BLAST) from the NCBI database and MEGA X.

### Molecular DNA characterization of *Strongyloides*

Three L3 larvae of *Strongyloides* were isolated from a positive charcoal culture of ‘clean-catch’ stool. The larvae were subjected to a boil and spin DNA extraction; briefly 50 *μ*L of TE/Proteinase K buffer [48 *μ*L TE and 2 *μ*L of proteinase K (20 mg *μ*L^−1^)] were added to each sample, then incubated at 56°C for 1 h followed by a final incubation at 93°C for 10 min.

For PCR-based identification of *Strongyloides*, 5 *μ*L of template DNA was used in a 25 *μ*L reaction with the remainder consisting of 12.5 *μ*L MyTaq™ Red Mix (Bioline), 400 nm of forward primer (5′-CCGATAACGAGAGAGACTTTTATG-3′), 400 nm of reverse primer (5′-GCCCGGTTCAAACAACAGCG-3′), adjusted using nuclease-free water. The genus-specific primer targeted a 18S sub-region that generated a 435 bp product. The thermal PCR cycle was initial denaturation at 95°C for 1 min, followed by 40 cycles of denaturation at 95°C for 15 s, annealing at 55°C for 15 s and extension at 72°C for 30 s. After the 40th cycle, there was an additional extension step of 72°C of 2 min. The DNA amplicons were separated in a 1.2% agarose gel for 1 h at 100 V and then stained with Invitrogen™ SYBR™ Safe DNA Gel Stain (Fisher Scientific).

Amplicons were excised from the gel and then cleaned using Exo-SAP™ IT (Thermofisher, Massachusetts, USA) following the manufacturer's protocol. DNA was sent for Sanger sequencing at Source Bioscience, UK using both forward and reverse primers. The obtained sequences were analysed using MEGA X. Sample sequences were then aligned against reference sequences of the same genus using the basic local alignment search tool (BLAST) from the NCBI database.

## Results

### Numbers of vehicles assessed during surveys

A total of 2662 vehicles were recorded driving through the baboon enclosure over the 4 surveys: Sa – 441, Sb – 809, Sc – 536 and Sd – 876. The average amount of time vehicles spent in the enclosure was 18 min 10 s (95% CI 17 min 38 s–18 min 32 s). Overall, the minimum time spent in the enclosure was 2 min and 30 s, with the maximum time being 52 min and 30 s.

**Figure 2.**
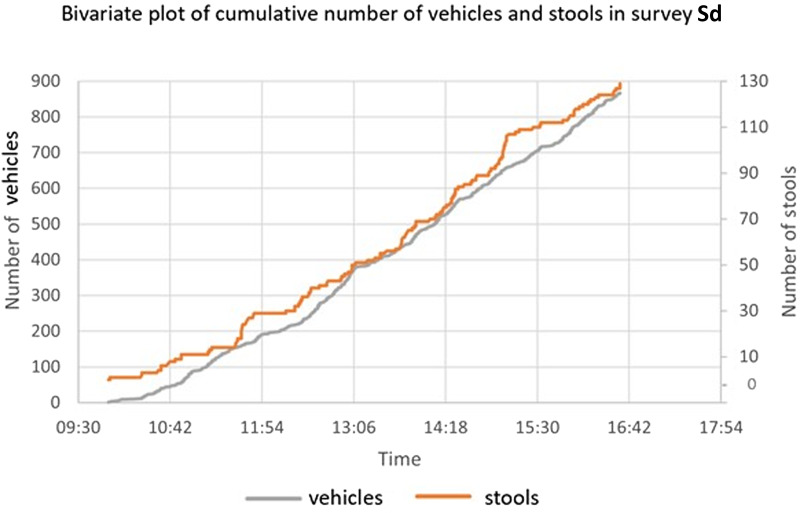
Bivariate plot of the cumulative number of vehicles plotted against the cumulative number of stools obtained throughout the day during survey Sd. The positive association follows a clear similar linear trend (vehicles *y* = 33 356.9*x* − 11 469.7; stools *y* = 515.0*x* − 228.5), with 1 or 2 exceptions, typically around times during or shortly after animal feeding.

The number of stools present on each vehicle and sleeping areas were recorded in each survey, see Table 1. Of all vehicles, 13.9% had at least 1 baboon stool on it upon exiting the enclosure. The most common vehicle's surface contaminated with stool was the roof, which comprised of 52.3% of all stools collected. Mid-week surveys Sa and Sc had a smaller percentage of vehicles contaminated by stool (11.3 and 9.5%) than weekend surveys Sb and Sd (13.6 and 12.6%) (Fig. 2). Overall, stool-contaminated vehicles had spent an average of 19 min and 46 s (95% CI 18 min 53 s–20 min 39 s) inside the enclosure, which was 3 min and 11 s longer than the average time of stool-free vehicles spent inside the enclosure.

**Table 1.** Prevalence of *Trichuris trichiura* and *Strongyloides fuelleborni* in baboon stools collected from vehicles and the sleeping areas across the 4-day (Sa, Sb, Sc and Sd) surveys

	Survey Sa	Survey Sb	Survey Sc	Survey Sd	Total
Stools from vehicles (*n*)	61	121	69	120	371
Stools from sleeping areas (*n*)	57	78	76	87	298
Stools from vehicles					
*T. trichiura* prevalence (%) 95% CI	33.6 CI: 13.9–53.3	33.7 CI: 12.4–36.4	28.4 CI: 23.4–36.3	44.9 CI: 29.4–48.5	35.2 CI: 28.3–34.2
*S. fuelleborni* prevalence (%) 95% CI	11.6 CI: 6.3–16.9	26.2 CI: 6.3–17.3	18.4 CI: 10.1–22.1	6.7 CI: 4.4–14.0	13.1 CI: 6.1–16.4
Stools from sleeping areas					
*T. trichiura* prevalence (%) 95% CI	81.1 CI: 43.2–119.0	66.2 CI: 32.8–122.5	38.1 CI: 26.3–45.7	63.2 CI: 44.6–73.0	62.5 CI: 36.3–58.2
*S. fuelleborni* prevalence (%) 95% CI	4.7 CI: 1.3–8.1	19.6 CI: 17.5–30.3	17.3 CI: 9.6–21.7	3.0 CI: 4.5–7.5	10.7 CI: 8.4–12.5

### Analysis of video footage of baboon–vehicle interactions

As video surveillance data from Sd was most complete, due to rapid battery recharge and good weather, it was used to investigate the baboon–vehicle interactions in more detail. Defecation on vehicles varied and depended on the time of day. It was noted that the time between 09:00–10:00 and 15:30–16:30 had fewer baboons defecating on the cars compared to between 11:30–12:30 and 13:30–15:30. Analysis of the footage captured by camera CamA recorded that the baboons were regularly fed by the keepers, across the 4 days of observation, around 11:00 am and 13:00 pm on each day.

The cumulative number of stools collected was plotted against the cumulative number of vehicles throughout the day in survey Sd in order to assess trends. Increases in stools were noted upon observation of the plot of gradient of the cumulative number of stools vs the number of vehicles around 11:30, 13:30 and 14:30. The increase in the cumulative number of vehicles entering the baboon enclosure appeared relatively constant across the day (Fig. 2).

### Incidental observations from video analyses

From perusal of all video footage and paper records, it was noted that several vehicles entered the baboon enclosure 2–3 times a day and a handful also recorded across the other 3 days of observations. In terms of baboon behaviour, more juvenile baboons (323) were observed climbing onto vehicles compared to adult female (226) and adult male (24) baboons. CamA, placed at the entrance/exit of the enclosure, typically recorded an increased number of baboons interacting with vehicles between 13:46 pm and 14:21 pm. Furthermore, around 11:00 am, baboons were fed at 1 location in view of this camera.

Simultaneously, CamB and CamC did not record baboons interacting with vehicles at this time when animals were focused on eating. Between 11:30 am and 11:50 am, and 12:48 pm and 14:35 pm, numerous baboons were seen climbing onto vehicles, consisting mainly of juveniles and their mothers, while adult male baboons were more likely to be seen foraging for food on the ground. There was a notable increase in the number of baboons, particularly adult males, on vehicles at 15:50 pm, when many cars had stopped moving at location A (most likely allowing visitors to take final pictures of the baboons as they exited). CamB recorded that there were more baboons interacting with vehicles at 12:24 pm and 13:39 pm. At these times, there were also increases in the number of queuing vehicles along the drive, ultimately exiting out of the enclosure, driving slowly at approximately 3 mph, under the supervision of keepers to remove any animals from the vehicles as they departed.

#### Front-line coprological results: QUIK-CHEK RDT

All stool samples collected from the sleeping area and cars were tested for *G. duodenalis* using the *Giardia*/*Cryptosporidium* QUIK-CHEK. The RDT showed the overall prevalence of giardiasis in the stool samples was 37.4% (95% CI 33.7–41.1); the prevalence between stools obtained from vehicles vs stools from sleeping areas was equivalent, at 37.4% (95% CI 32.2–43.3) and 37.0% (95% CI 32.0–42.0), respectively (*Z*-score = −0.08, P = 0.93). A serial dilution of 2 very strongly positive samples estimated that the concentration of coproantigens would infer some 6 × 10^6^ cysts per g of stool. Although 2 RDTs returned positive results for *Cryptosporidium*, inferring a prevalence of less than 0.01% within the sampled stools, cysts could not be confirmed upon microscopy, thus an absence of cryptosporidiosis was inferred.

#### Front-line coprological results: Kato–Katz

**Figure 3.**
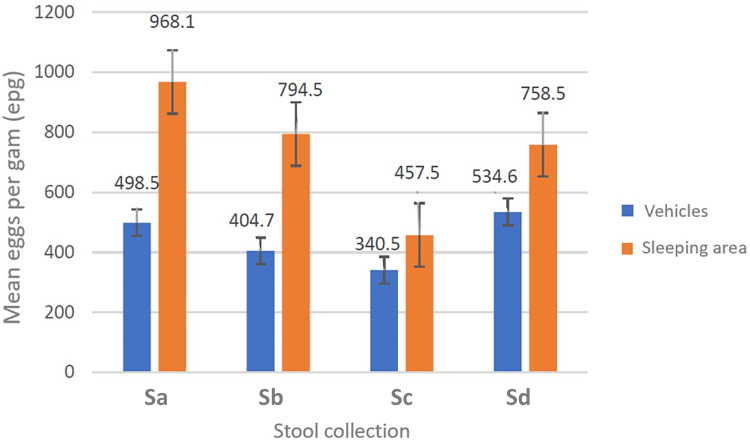
Intensity of infection for *Trichuris trichiura* across surveys (Sa, Sb, Sc and Sd) and sampling methods (from vehicles or from sleeping area) with geometric mean EPG indicated. The geometric mean EPG for sleeping area stool was some 2-fold higher than that from vehicles across all 4 collections.

A double Kato–Katz slide was examined for all stool samples (*n* = 669). The total prevalence of *T. trichiura* was 48.0% across both the vehicles (*n* = 371) and 2 sleeping areas (*n* = 298) but the prevalence between the stools obtained from vehicles and stools from sleeping areas was not equivalent, at 35.2% (95% CI 30.2–40.1) and 62.5% (95% CI 56.0–67.3), respectively (*Z*-score = −7.22, P < 0.0001. From the vehicle surveys, the highest parasite prevalence was in Sd (44.9%) while the lowest was in Sc (28.4%); in the sleeping areas, the highest prevalence was in Sa (81.1%) while the lowest was in Sc (38.1%). Of note and following the WHO guidelines (WHO, 2011), the intensity of all *T. trichiura* infections was deemed *light* (⩽999 EPG) (see Table 1). The mean EPG for each day of survey, and by collecting method, is plotted in Fig. 3; the highest mean EPG of 968.1 was noted from survey Sa and from sleeping area stool. No other ova of helminths were observed, with the exception of a single-morulated egg within a single Kato–Kato smear.

#### Front-line coprological results: charcoal culture

The prevalence of *Strongyloides* in stool samples from vehicles was 13.7% (95% CI 5.9–17.8) and from sleeping areas was 10.7% (95% CI 4.8–18.5), *Z*-score = 0.65, P = 0.52. From the vehicle collection, the highest prevalence was found in Sb (26.2%) and the lowest prevalence of 6.7% in Sd. In the sleeping areas, the highest prevalence was found in Sb (19.6%) and the lowest in Sd (3.0%).

#### Second-line coprological results

For each sample preparation a total of 6 slides were examined, while ova of *Trichuris* were frequently seen, neither cysts of *Giardia* were noted nor any other notable parasites seen.

### Observations on DNA typing

PCR amplification of a 511-bp region of the *G. duodenalis bg* gene was mostly unsuccessful, although a sequence was obtained from 4 samples which, upon a BLAST search of GenBank, most closely matched a *G. duodenalis* assemblage B lineage [MF581553.1 (coverage: 100%; identity score: 99.01%; *E*-value: 8.00 × 10^−155^)]. This lineage originated from children from Western Angola.

When aligned against the NCBI database our *Trichuris* sequence was found to have a 100% query cover and identity to 3 reference sequences for *T. trichiura* (MF288624, MF288618 and LC596914). The closest, non-*T. trichiura*, sequence match was *Trichuris suis* with a query cover of 100% and identity value of 97.36% (MF288628). The bootstrapped maximum-likelihood tree clearly placed our sequence within the *T. trichiura* clade.

As 2 of the 3 18S sequences for *Strongyloides* from Knowsley were identical, 2 sequences, differing by a single (C/A) polymorphism are deposited in GenBank (accession numbers: OR395366 and OR395367), finding closest matching species by BLAST to be *Strongyloides fuelleborni fuelleborni*, with 100% query cover and 99.08% identity (AB272235) followed by *Strongyloides papillosus*, query cover 100%, identity 98.62% (AB923886) and *Strongyloides venezuelensis*, query cover 100%, identity 97.7% (LM524969).

## Discussion

Our coprological investigation clarified and quantified the occurrence of gastrointestinal parasites of public health importance within a UK colony of captive olive baboons, open to the public *via* vehicle safari. Our survey design took advantage of seminal information from Lloyd *et al*. (2021) who documented general vehicle movements and animal activities, across several KS drive-through areas. Vehicle-faecal contamination rates were relatively stable across the 4 survey days: Sa (13.8%), Sb (14.9%), Sc (12.8%) and Sd (13.7%), with an overall contamination rate of 13.9%. With 1 or 2 exceptions, around animal-feeding times, the accumulation of contaminating stools on vehicles throughout the day broadly followed a linear relationship, Fig. 2, a common trend, most likely repeated throughout other times of the year.

### Baboon–vehicle interactions

By slow-motion viewing, analysis of our remote video surveillance footage revealed juvenile baboons were more likely to mount vehicles than adults (1.3:1.0), as were adult females compared to adult males (9.4:1.0). These behavioural biases reflect perhaps the more inquisitive nature of younger baboons and closer supervision of their movement by their matriarchs and kinship groups. However, when vehicles were queuing to enter or exit the enclosure, baboons were more likely to mount vehicles as they could easily walk from parked vehicle-to-vehicle which in turn increased vehicle contact times alongside opportunities for defecation. Similarly, when baboons were being fed by their keepers, some visitors stopped driving to watch the baboons eating, thus the keepers’ activities, either directly or indirectly, influenced baboon defecation behaviour.

### Parasites of public health importance

To gain best appraisal as guided by diagnostics selected from our pilot investigation, we examined 371 ‘clean-catch’ stools from vehicles alongside 298 ‘ground-picked’ stools from sleeping areas, a total of which is just under 3 times the total number of animals present within this enclosure. Assuming some duplication bias between collection strategies, it might be reasonable to infer parable estimations of the ‘true’ infection prevalence in the colony; hence, our recommendations have wider bearings for managing baboons in zoological collections. We review our most salient findings, discussing their wider implications.

#### On giardiasis

We considered that use of QUIK-CHEK RDT, as we have used in the past for human analyses (Al-Shehri *et al*., 2016) that has good diagnostic sensitivity and specificity (Van den Bossche *et al*., 2015), would perform equally well for detection of coproantigens of *Giardia* within baboon stool. Of note, this RDT was also evaluated by Saleh *et al*. (2017) in their study of a ‘pathogen-free’ captive olive baboon colony, obtaining favourable results. Upon face value here, prevalence of *Giardia* from ‘clean-catch’ vs ‘ground-picked’ stool was essentially the same, at 37.4%, which is intriguing when set against known baboon–vehicle interactions (see above) and our prevalence and intensity observations of trichuriasis (see below).

A cursory serial dilution series analysis, with repeated QUIK-CHEK RDT testing, of 2 very strongly positive stools indicated coproantigen levels would be approaching 6 × 10^6^ cysts per g of stool yet quality control coproscopy did not yield cysts of *Giardia* and PCR analysis failed to confirm high levels of *Giardia* DNA. Taken as a whole, we conclude that our RDT results were misleading and were likely artefactual. We surmise that RDTs have grossly overestimated the prevalence of giardiasis in the colony. Such false positives were resultant perhaps from baboons ingesting ubiquitous, then excreting cross-reacting environmental antigens that interfered with the QUIK-CHEK RDT.

#### On trichuriasis

From molecular DNA analysis of adult worms obtained at necropsy of 2 baboons in 2019 and then in 2021 (unpublished), we confirmed the presence of the human whipworm *T. trichiura*. From Kato–Katz coproscopy, the prevalence of infection in ‘clean-catch’ vs ‘ground-picked’ stool was consistently different, 35.2% (95% CI 28.3–34.2) vs 62.5% (95% CI 36.3–58.2). While all ova patent infections were classified as *light intensity*, the average EPG of ‘ground-picked’ stool was around 2-fold higher, Fig. 3. Nevertheless, it is reasonable to assume that the prevalence of trichuriasis is close to 50.0% within the colony. Anderson *et al*. (2012), Eo *et al*. (2019) and Larbi *et al*. (2020) have each noted whipworm infections in baboons previously, and whipworm control in people is a major ongoing effort across the world (WHO, 2012).

Following from our video analyses, this consistent difference between collection methods might reflect a decreased prevalence and reduced infection intensity in more juvenile animals, and their female matriarchs, vs all others in the colony. Nonetheless trichuriasis is clearly highly endemic here and is of some public health concern. Safe disposal of baboon stool on vehicles, alongside regular change and prompt incineration of baboon bedding, is important. It should be remembered, however, that any immediate ingestion of recently excreted *Trichuris* ova is not hazardous, owing to an insufficient period of external environmental development under favourable conditions (Manz *et al*., 2017). With appropriate precautions, any risk to the public, or KS staff, of ingestion of large numbers of infectious ova is negligible.

To diminish the prevalence of infections within the colony we can draw firm parallels with current preventive chemotherapy approaches against human trichuriasis. These are now moving towards combined oral treatment of ivermectin and albendazole (Patel *et al*., 2019), owing to greater anthelminthic tolerance of *T. trichiura*. This might have some later consequence in choice of medicines in future blanket deworming of the colony, alongside fenbendazole (Reichard *et al*., 2017), as future medications are distributed (see below).

#### On strongyloidiasis

From charcoal culture, the prevalence of strongyloidiasis was 13.7% from the ‘clean-catch’ stool and 10.7% from ‘ground-picked’ stool. Human strongyloidiasis is of particular global public health concern, and the main human threadworm species responsible is *Strongyloides stercoralis* (Olsen *et al*., 2009). From DNA inspections and from later charcoal cultures specifically conducted to obtain adult female worms, Fig. 1E, enabling morphological differentiation upon vulvar features (Muller, 2002), we can carefully exclude this species here and confidently note the occurrence of the threadworm *S. fuelleborni*.

*Strongyloides fuelleborni* is a common parasite of non-human primates and has been found within baboon colonies before (Anderson *et al*., 2012). Of note, it has also been proven by experimentation to infect people (Pampiglione and Ricciardi, 1972). This species has a slightly different lifecycle from *S. stercoralis*, being unable to autoinfect its host and embryonating eggs, rather than larvae, are expelled in the stool (Muller, 2002). Any percutaneous exposure(s) to L3 larvae, later emergent from baboon stool or within the baboon enclosure's soils, will carry a tangible public health risk of zoonotic strongyloidiasis.

As for trichuriasis, safe disposal of baboon stool on vehicles alongside regular change and prompt incineration of baboon bedding is particularly important for mitigation of strongyloidiasis. Furthermore, when cleaning older animal bedding or soiled materials, extra care should be taken not to expose skin. If so, immediate post-exposure application of ethanol-based disinfectant hand gels, now commonly available since COVID-19, should be an efficacious knock-down of any adhered larvae. Given the importance of zoonotic strongyloidiasis, any baboon to be transferred away from KS should be carefully screened for *S. fuelleborni*, dewormed with most efficacious anthelminthic combination, then re-screened until negative before any later movement(s) are put in place.

Currently, best medications against strongyloidiasis are being debated. Human trials are exploring oral ivermectin and albendazole combinations (Patel *et al*., 2019) as well as ivermectin and fenbendazole in baboons (Reichard *et al*., 2017). Additionally, the future use of emodepside could be considered given its recent favourable laboratory evaluation against a wide range of gastrointestinal nematodes (Karpstein *et al*., 2019). From past experience at KS, oral administration of medications to baboons in food has not been judged successful, hence a ‘pour-on’ anthelminthic formulation, using partial animal restraint, could be most pragmatic to distribute medicines *en masse* with current amenities.

## Conclusion

From a public health perspective, our coprological investigation of KS baboon colony has highlighted an absence of cryptosporidiosis, inconsequential giardiasis, but has very clearly confirmed the infection dominance of trichuriasis alongside the strong presence of zoonotic strongyloidiasis. Resolving the phylogeographical origin(s) of *T. trichiura* and *S. fuelleborni* reported here requires inspection of additional DNA loci. Nevertheless, to diminish current levels of helminthiases, we recommend regular blanket administration of anthelminthics, exploring pour-on formulations, thereafter smaller-scale parasitological indicator surveys would be sufficient to assess any further public health concerns.
